# How do muscle injuries relate to return-to-performance metrics in male elite football players?

**DOI:** 10.5114/biolsport.2026.153532

**Published:** 2025-08-13

**Authors:** Marc Guitart, Antonio Alonso-Callejo, Gil Rodas, Francesc Cos, Andres Martin-Garcia, Xavi Franquesa, Berta Carles, Xavier Valle, Xavier Yangüas, Jose Luis Felipe

**Affiliations:** 1 FC Barcelona Performance Department, Barcelona, Spain; Barça Innovation Hub, Health & Wellness Area, Barcelona, Spain; 2 Universitat Autònoma de Barcelona, Barcelona, Spain; 3 Department of Sports Sciences, Faculty of Medicine, Health and Sports, Universidad Europea de Madrid, Madrid, Spain; 4 FC Barcelona Medical Department (FIFA Medical Center of Excellence), Barcelona, Spain; 5 Barça Innovation Hub, Health & Wellness Area, Barcelona, Spain; 6 Leitat Technological Center, Terrassa, Spain; 7 Manchester City Football Club 1st Team, Manchester M11 4TS, UK; 8 National Institute of Physical Education of Catalonia (INEFC), University of Barcelona, 08038, Barcelona, Spain; 9 ICATME-DEIXEUS: Unidad Medicina del Deporte, 08028, Barcelona, Spain; 10 IGOID Research Group, Department of Physical Activity and Sport Sciences, University of Castilla-La Mancha, 45071, Toledo, Spain; 11 Performance Analysis Department, UD Las Palmas, 35019, Las Palmas de Gran Canaria, Spain

**Keywords:** Rehabilitation, Return-to-play, Elite football, Training load

## Abstract

The aim of this study was to analyze how the type, location, and severity of injury are associated with the time elapsed since the return to training and matches of male football players to reach fitness values comparable to pre-injury levels. A longitudinal analysis was conducted on 333 male football players from an elite Spanish football club over five seasons (2017/18 to 2021/22). A total of 234 injuries (including medical attention) were included in the analysis. The study focused on hamstring and quadriceps injuries, examining high-speed running, sprint distance, and maximum velocity before and after injury. Results indicated that hamstring injuries significantly impact V_max_ and HSR, with severe injuries requiring up to five weeks to return to pre-injury levels (V_max_: −1.43 km/h, p = 0.01; HSR: −32.90 m, p = 0.04). Quadriceps injuries revealed less impact on performance metrics than hamstring injuries, with only mild injuries resulting in a significant reduction in V_max_ (−1.18 km/h, p = 0.04) and HSR (−52.70 m, p = 0.01) during the first week post-injury. The findings highlight the importance of injury-specific rehabilitation protocols and the need for tailored training loads to minimise the risk of re-injury and optimise return to performance. This research provides valuable insights for medical and performance staff in elite football, emphasizing the critical role of injury management in maintaining player performance and club success.

## KEYPOINTS

– Hamstring injuries in professional football players significantly impact their performance, particularly in high-speed running and maximum velocity, which can take up to five weeks to recover to pre-injury levels.
– Quadriceps injuries show a different recovery pattern, with less impact on performance metrics compared to hamstring injuries, highlighting the importance of injury location in recovery and performance planning.
– The study emphasizes the need for coordinated rehabilitation and performance strategies to ensure players return to their pre-injury performance levels effectively and safely.

## INTRODUCTION

Football is an intermittent sport that combines high-speed actions with longer periods of walking and jogging [1]. High-speed actions are crucial in determining the success of this sport as they often precede goal situations [2]. The quadriceps play a significant role in sprinting, jumping, and ball kicking [3], while the hamstrings assist the anterior cruciate ligament in preventing anterior draw forces and decelerating the leg prior to full extension, thus limiting knee overextension [4].

Muscle injuries of the lower extremity are the most common injuries in elite football, representing about one-third of all time-loss injuries [5]. Among these, hamstring injuries are the leading cause of lost playing time, accounting for 12–17% of all football-related injuries in the 2014/15 season and up to 24% in the 2021/22 season, with time-loss varying from days to several months [6]. Between 2014/15 and 2021/22, the incidence of training-related hamstring injuries increased by 6.7% annually, and the burden increased by 9.0%. During the same period, the incidence of match-related hamstring injuries increased by 3.9% annually [6].

At the elite club level, injury incidence is 6.1 per 1000 hours overall, 19.2 per 1000 hours in matches, and 3.5 per 1000 hours in training [7]. Injuries have a significant economic impact on elite clubs, with the cost of an elite football player being absent for one month estimated at €500,000 [8]. Additionally, team success is correlated with greater player availability and a lower rate of injuries [9]. Therefore, implementing preventive measures to reduce football injuries has received considerable attention from sports physical therapists [10]. The challenge for medical and physical coaching staff is to ensure a quick return to pre-injury performance levels with minimal risk of re-injury [11].

While research has focused on treatment protocols and the early phases before return to play (RTP) [11], there is limited research on the period from RTP to return to performance (RTPerf), defined as the time required to achieve pre-injury performance levels or higher [12]. Previous studies have analyzed the return to performance after hamstring injuries [13–15], focusing on GPS external load regarding distance traveled at different intensities and maximal velocity to determine changes in match-based physical performance parameters before and after sustaining a hamstring strain injury.

Studies have shown a performance decrease in high-intensity distance running up to 15 matches after RTP [14] and a reduction in maximal velocity two months after RTP during a 50 m sprint test [16]. In contrast, other studies have shown a faster improvement, with 15 out of 18 injuries showing a return to pre-injury maximal velocity in the second match after RTP [15], and likely improvements reported for all match load parameters at the moment of RTP and 6–10 weeks after RTP compared with pre-injury performance [17]. Understanding the context of muscle injury incidence in training programs and official games is critical for making the best decisions in RTP and RTPerf criteria. However, the location of the injury directly affects the physical demands achieved when returning to play.

Therefore, the aim of this study was to analyze how the type, location, and severity of injury are associated with the time elapsed since the return to training and matches of male football players to reach fitness values comparable to pre-injury levels.

## MATERIALS AND METHODS

### Participants and study design

A descriptive and longitudinal prospective study was developed with a total of 333 elite-football players (Table 1) in three different teams (male 2^nd^ Team, male U-19 team, and male U-18 team) from an elite football club in Spanish La Liga that also competed in the Union of European Football Associations (UEFA) Champions League during 5 seasons, from 2017/18 to 2021/22 (Table 1).

**TABLE 1:** Descriptive participant data.

Team	Players (n)	Age (years)	Weight (kg)	Height (cm)
**Season 2017/18**					
Male 2^nd^ Team	34	20.85 ± 2.25	73.70 ± 5.74	179.71 ± 6.77
Male U-19	17	17.85 ± 0.48	68.17 ± 6.72	176.50 ± 7.50
Male U-18	19	16.60 ± 0.63	66.17 ± 8.16	176.10 ± 6.91

**Season 2018/19**					
Male 2^nd^ Team	30	19.71 ± 1.19	71.89 ± 7.19	179.05 ± 6.91
Male U-19	22	17.71 ± 0.79	70.35 ± 6.00	179.35 ± 6.13
Male U-18	25	16.58 ± 0.59	66.77 ± 7.90	176.57 ± 6.70

**Season 2019/20**					
Male 2^nd^ Team	31	19.85 ± 1.76	72.46 ± 6.79	179.06 ± 5.70
Male U-19	19	17.95 ± 0.64	70.22 ± 6.22	179.16 ± 7.12
Male U-18	18	16.60 ± 0.52	65.70 ± 8.01	176.57 ± 7.57

**Season 2020/21**					
Male 2^nd^ Team	21	20.19 ± 1.70	73.04 ± 5.43	179.79 ± 5.37
Male U-19	18	17.61 ± 0.87	70.00 ± 6.26	175.87 ± 5.41
Male U-18	21	16.59 ± 0.49	64.74 ± 5.44	170.00 ± 5.31

**Season 2021/22**					
Male 2^nd^ Team	22	20.46 ± 1.80	71.09 ± 6.98	177.96 ± 6.17
Male U-19	20	17.92 ± 0.57	70.40 ± 7.81	176.38 ± 5.59
Male U-18	16	16.62 ± 0.47	67.53 ± 7.51	174.35 ± 6.87

Mean ± standard deviation (SD).

All players belonging to the squads of one of the three teams of each season were eligible for inclusion. Players who were transferred to other clubs or who finished their contracts due to other reasons before the end of a season were included for as long as they trained and played for the club [18]. The goalkeepers were excluded from the study. Informed consent was obtained from all participants, including those under the age of 18. For minors, written informed consent was provided by their legal guardians, in accordance with the ethical standards outlined in the Declaration of Helsinki. This procedure was approved by the Ethics Committee of FC Barcelona (approval number: 2019FCB28).

### Procedures

#### Description of structured microcycle training model

A total of 161,280 h of training sessions (TSs), 18,270 h of friendly games (FGs), and 60,120 h of official matches (OMs) were analyzed. Data were collected from U-18 (807 TSs, 82 FGs, and 220 OMs), U-19 (881 TSs, 73 FGs, and 216 OMs), and 2^nd^ Team (1,000 TSs, 48 FGs, and 232 OMs).

All three teams shared the same training methodology and game model. The training methodology was the structured microcycle [19], in which football content structuration was usually cyclical, but the external load oscillated based on the objectives of each seasonal stage. The most common microcycle structure was as follows: the first day after the match, the training load was focused on recovery training for the players who played > 60 min the day before or a performance compensatory training to achieve the workload missed by not having played the match or having participated less than 60 min. According to the context of some weeks, this session could be two days after the match. After a rest day, the training load was progressively increased up to three days before the next match, progressively decreasing the load until the day of the next match [20].

### Quantifying workload

External load was quantified using GPS data collected from all onpitch TSs and matches during all season phases. Each player was assigned their own specific device to avoid potential inter-unit reliability error. GPS data were collected using the WIMU PRO device (RealtrackSystems S.L., Almeria, Spain). Intra- and inter-unit reliability was acceptable (the intra-class correlation coefficient value was 0.65 for the × coordinate and 0.85 for the y coordinate) for the systems analyzed [20]. The data collected were analyzed using the SPRO Software (v982, v983, and v984; RealtrackSystems S.L., Almeria, Spain), which exports the data in raw format.

For the main section of this study, variables related to high intensity (HSR distance, Sprint distance, and V_max_) were included. The remaining analyzed variables were provided in the supplementary files for additional information for the readers and to present the results more clearly. The speed thresholds used in this study were: High-Speed Running (HSR) defined as running above 21 km/h, and Sprinting defined as running above 24 km/h, in accordance with thresholds commonly used in elite football performance analysis [21].

### Muscle injury recording

This study is based on consensus guidelines for the definitions and data collection procedures for football injury studies described by the UEFA [22]. The injuries were classified according to the severity [23] and based on UEFA proposal according to the number of days from injury occurrence until the end of medical leave, ranging from medical attention (0 days), mild (1–7 days) to moderate (8–28 days), and severe (> 28 days) [22, 5]. Injuries had to occur during training or a match and cause medical attention or the player to be absent from at least the following TS or match. Injury was defined, using a time-loss definition, as ‘any physical complaint sustained by a player that results from a football match or football training and led to the player being unable to take full part in future football training or match play’ [22].

Injuries were classified using the OSICS_10 coding system (Orchard Sports Injury Classification System), and muscle injuries were indicated by the code OSICS -M--. This study analyzed muscle injuries of the lower limbs, specifically hamstrings and quadriceps. Injuries that did not show visible damage in diagnostic images but still prevented the player from participating were also included and termed as medical attention injuries. Free tendon injuries were excluded due to the difficulty in obtaining consistent and reliable data on this type of injury in the context of professional football. All diagnoses were made by the same medical staff throughout the study period, using imaging support (ultrasound or magnetic resonance imaging) to ensure consistency and accuracy. The criteria for diagnosis, prognosis and treatment up to the RTP were following the FC Barcelona muscle guide [21].

**FIG. 1:**
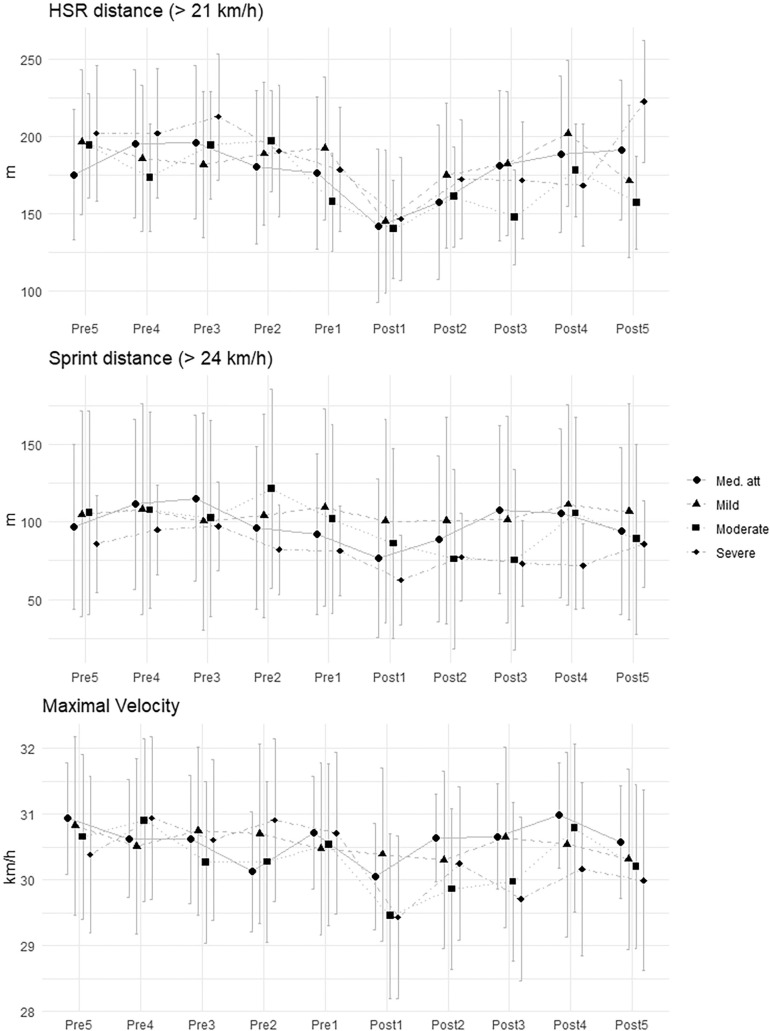
Mean and standard deviation of external load for each time window in hamstring injuries.

**FIG. 2:**
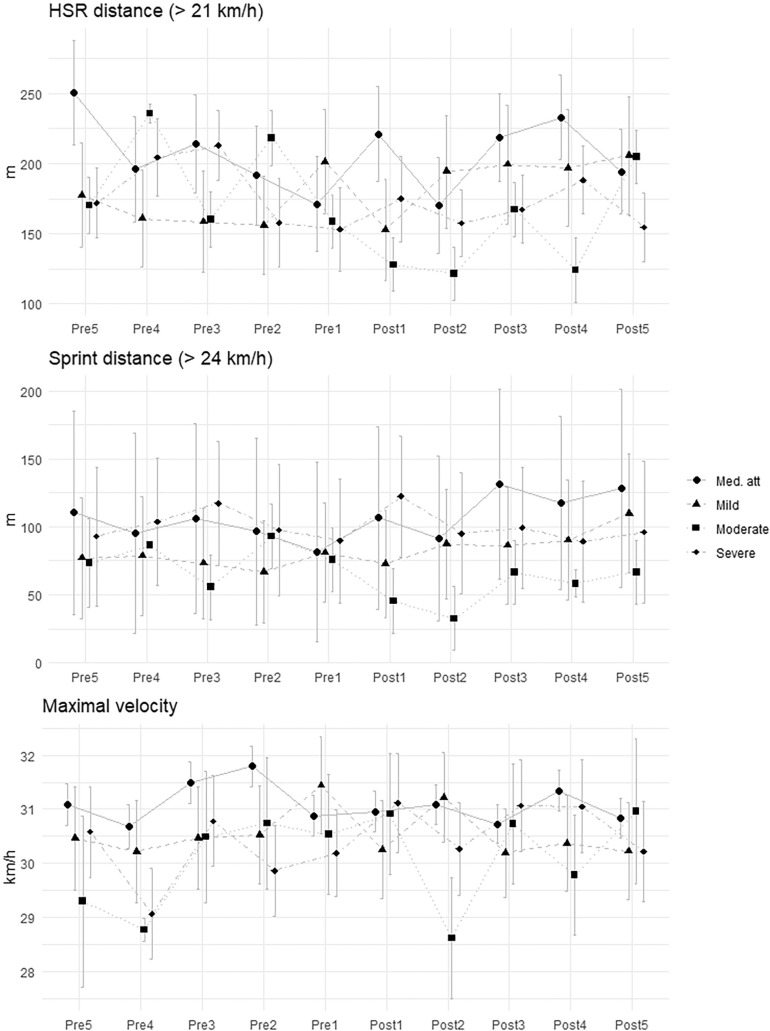
Mean and standard deviation of external load for each time window in quadriceps injuries.

To establish time windows, the day of the injury was taken as the reference day. From this day, 7-day periods prior to the injury were established and are referred to with the prefix Pre, followed by a number corresponding to the specific time window before the injury. Thus, the period Pre1 encompasses the day before the injury to the 7^th^ day prior to the injury, the period Pre2 from the 8^th^ day prior to the injury to the 15^**th**^ day, Pre3 from the 16^th^ day to the 23^rd^ day, Pre4 from the 24^th^ day to the 31^st^ day, and Pre5 from the 31^st^ day to the 38^th^ day. These 7-day periods were established to ensure that each time window included at least one match and, in the case that the player had not participated, to include MD+1 where loads like competition are reached.

Similarly, the day the injured player completed their first training session with the team or participated in an official match for at least 60 minutes was taken as the reference day for RTP. From this point, time windows were established in the same manner but were referred to as Post. Thus, Post1 corresponds to the reference day mentioned above until the 7^th^ day after it, Post2 from the 8^th^ day to the 15^th^ day, Post3 from the 16^th^ day to the 23^rd^ day, Post4 from the 24^th^ day to the 31^st^ day, and Post5 from the 31^st^ day to the 38^th^ day. To avoid bias with the number of TS included in each time window, the analysis was developed using the average load of that period for HSR, Sprint, and V_max_ reached within the time window. Furthermore, at least two maximal sprints were performed in each time window to ensure maximal values were achieved independently of the requirements of competitions and training.

Five windows were used for pre- and post-injury follow-up because extending the follow-up period significantly reduced the number of players with complete data. Players without data in any follow-up period were excluded from the study.

### Statistical Analysis

A linear mixed model (LMM) was developed in which physical variables (HSR, Sprint, and V_max_) were the dependent variables for the models. One model was performed for each muscular region and injury severity. The predictor variables with fixed effects included for each model were: Time Windows (as factor and with ten levels Pre5, Pre4, Pre3, Pre2, Pre1, Post1, Post2, Post3, Post4, Post5, being Pre1 as the reference level), Days Off (numeric). For injuries classified as requiring medical attention, the variable “Days Off” was not included because it was always 0. The model accommodates the hierarchical structure of the data, where multiple observations (training sessions and matches) are nested within individual players. This approach helps to control for the variability between players and provides more accurate estimates of the fixed effects. All statistical analyses were performed in R version 4.2.2 (The R Foundation for Statistical Computing, Vienna, Austria), with RStudio 2022.12.0 using the functions lmer() from the package lme4 (version 3.1–162) for the model fit.

## RESULTS

A total of 248 injuries (81 medical attention and 167 time loss) were included in the analysis (Table 2). The results of the LMM for each injury region and severity are shown in Tables 3 and 4. The intercept of the model corresponds to the Pre1 stage, which was set as the reference level to enable meaningful comparisons.

**TABLE 2:** Descriptive injuries data.

Muscle	Severity	n	Time loss (days)	Injury burden
Quadriceps	Med. Att.	28	0.0 ± 0.0	0.0
	Mild	21	4.1 ± 1.4	12.3
	Moderate	8	12.2 ± 3.1	7.0
	Severe	10	77.8 ± 26.9	111.0

Hamstrings	Med. Att.	53	0.0 ± 0.0	0.0
	Mild	38	3.5 ± 1.4	19.0
	Moderate	31	16.5 ± 6.9	49.6
	Severe	59	128.0 ± 105.2	913.0

Time loss days (mean ± standard deviation [SD]); Med. Att = medical attention. Injury burden denotes number of injury days absence divided by the total exposure time (h) × 1000.

For hamstring injuries (Table 3), the LMM revealed significant reductions (p < 0.05) in HSR during Post1 period for all types of injuries except moderate (medical attention: -34.37, SE = 15.83; mild: -45.81, SE = 18.64; severe: -32.90, SE = 16.78). It is important to highlight that V_max_ significantly decreases after the injury, especially in severe injuries (Post1: -1.43, SE = 0.36; Post2: -0.73, SE = 0.38; Post3: -1.16, SE = 0.39; Post4: -0.73, SE = 0.39; Post5: -0.90, SE = 0.40).

**TABLE 3:** Linear mixed model for hamstrings in male football players.

		HSR (m)			Sprint (m)			V_max_ (km/h)		

		Value	SE	*p*	Value	SE	*p*	Value	SE	*p*
**Medical Attention (n=53)**	(Intercept)	178.16	13.95	< 0.01	94.22	12.15	0.00	30.78	0.33	< 0.01
	Pre5	-8.04	16.78	0.63	1.14	12.10	0.93	0.18	0.42	0.67
	Pre4	19.37	16.46	0.24	16.63	11.85	0.16	-0.12	0.42	0.78
	Pre3	22.61	16.57	0.17	26.11	11.80	0.03^*^	-0.05	0.41	0.90
	Pre2	4.52	15.91	0.78	5.63	11.33	0.62	-0.55	0.40	0.17
	Post1	-34.37	15.83	0.03^*^	-16.78	11.37	0.14	-0.71	0.40	0.07
	Post2	-15.70	16.46	0.34	-6.69	11.86	0.57	-0.22	0.42	0.60
	Post3	9.45	16.57	0.57	8.31	12.03	0.49	-0.06	0.42	0.89
	Post4	15.87	16.67	0.34	6.47	12.20	0.60	0.24	0.43	0.57
	Post5	13.22	16.67	0.43	-0.84	11.86	0.94	-0.14	0.42	0.73

**Mild injury (n=38)**	(Intercept)	167.87	27.31	< 0.01	113.67	25.17	0.00	29.48	0.69	< 0.01
	Pre5	-0.62	19.45	0.97	-7.61	12.28	0.54	0.22	0.43	0.61
	Pre4	-11.85	19.64	0.55	-4.24	12.41	0.73	-0.19	0.43	0.67
	Pre3	-8.12	19.64	0.68	-12.78	12.52	0.31	0.25	0.43	0.57
	Pre2	-6.92	19.12	0.72	-5.00	12.08	0.68	0.17	0.42	0.69
	Post1	-45.81	18.64	0.01^*^	-9.06	11.95	0.45	-0.02	0.42	0.95
	Post2	-18.68	19.84	0.35	-10.52	12.68	0.41	-0.10	0.44	0.82
	Post3	-11.39	19.47	0.56	-5.97	12.43	0.63	0.21	0.43	0.63
	Post4	6.91	19.84	0.73	6.80	12.55	0.59	0.14	0.43	0.75
	Post5	-20.92	20.50	0.31	-4.93	12.96	0.70	-0.02	0.45	0.97
	Days_off	5.95	6.38	0.35	-0.68	5.94	0.91	0.26	0.17	0.12

**Moderate injury (n=21)**	(Intercept)	145.48	28.99	< 0.01	91.79	23.43	0.00	30.56	0.66	< 0.01
	Pre5	37.47	27.21	0.17	1.03	15.33	0.95	0.30	0.53	0.57
	Pre4	16.52	27.65	0.55	8.15	15.58	0.60	0.46	0.54	0.39
	Pre3	37.54	27.65	0.18	3.00	15.58	0.85	-0.18	0.54	0.74
	Pre2	41.15	26.79	0.13	14.41	15.33	0.35	-0.12	0.53	0.82
	Post1	-17.52	26.05	0.50	-15.64	14.65	0.29	-1.00	0.51	0.04^*^
	Post2	3.43	26.39	0.90	-20.71	14.84	0.16	-0.68	0.51	0.19
	Post3	-12.65	26.75	0.64	-24.07	15.05	0.11	-0.64	0.52	0.22
	Post4	17.74	26.39	0.50	-1.17	15.07	0.94	0.24	0.54	0.65
	Post5	-3.20	26.39	0.90	-15.60	14.85	0.29	-0.32	0.51	0.53
	Days_off	0.75	1.27	0.56	0.57	0.95	0.55	0.00	0.03	0.95

**Severe injury (n=59)**	(Intercept)	183.44	15.31	< 0.01	80.05	8.72	0.00	31.09	0.35	< 0.01
	Pre5	16.99	18.68	0.36	-0.81	9.86	0.94	-0.66	0.40	0.10
	Pre4	20.70	17.39	0.23	12.59	9.16	0.17	0.05	0.37	0.90
	Pre3	30.63	17.29	0.08	15.06	9.06	0.10	-0.27	0.36	0.46
	Pre2	10.02	17.39	0.56	0.43	9.11	0.96	0.00	0.37	0.99
	Post1	-32.90	16.78	0.04^*^	-20.27	8.96	0.02^*^	-1.43	0.36	0.01^*^
	Post2	-10.74	17.70	0.54	-6.27	9.51	0.51	-0.73	0.38	0.04^*^
	Post3	-13.63	18.04	0.45	-10.63	9.51	0.26	-1.16	0.39	0.01^*^
	Post4	-16.80	18.41	0.36	-10.07	9.80	0.30	-0.73	0.39	0.04^*^
	Post5	34.52	18.99	0.07	3.88	9.95	0.70	-0.90	0.40	0.02^*^
	Days_off	-0.08	0.06	0.18	-0.03	0.03	0.30	0.00	0.00	0.01^*^

Time windows represent 7-day periods relative to the injury. “Pre” periods refer to the weeks before the injury (e.g., Pre1 = 0–7 days before), and “Post” periods refer to the weeks after return to play (e.g., Post1 = 0–7 days after RTP). The intercept represents the Pre1 time window for a medical attention injury with 0 days of time loss. Metrics include High-Speed Running (HSR, > 21 km/h), Sprint Distance (> 24 km/h), and Maximum Velocity (V_max_, in km/h). Values are presented as mean ± Standard Error (SE).

For quadriceps injuries (Table 4), the LMM revealed significant decreases in HSR (-52.70, SE = 19.71) and V_max_ (-1.18, SE = 0.59) during the Post1 period for mild injuries. Additionally, there were significant decreases in sprint distance until the Post2 period for moderate injuries (-43.09, SE = 18.81).

**TABLE 4:** Linear mixed model for quadriceps in male football players.

		HSR (m)			Sprint (m)			V_max_ (km/h)		

		Value	SE	*p*	Value	SE	*p*	Value	SE	*p*
**Medical Attention (n=28)**	(Intercept)	175.66	19.67	< 0.01	90.41	19.02	0.00	30.89	0.38	< 0.01
	Pre5	76.13	26.53	0.01^*^	18.97	17.01	0.27	0.16	0.55	0.77
	Pre4	23.17	26.53	0.38	3.60	15.46	0.82	-0.18	0.56	0.75
	Pre3	41.18	25.51	0.11	18.37	14.84	0.22	0.61	0.54	0.26
	Pre2	20.32	25.21	0.42	10.88	14.63	0.46	0.95	0.53	0.07
	Post1	47.87	24.93	0.06	23.85	14.35	0.10	0.08	0.51	0.88
	Post2	-1.07	24.68	0.97	16.70	14.20	0.24	0.19	0.52	0.71
	Post3	51.14	25.20	0.04^*^	46.55	14.86	0.01^*^	-0.10	0.53	0.85
	Post4	62.49	25.80	0.01^*^	41.08	14.86	0.01^*^	0.50	0.53	0.34
	Post5	25.16	25.80	0.33	38.23	15.50	0.01^*^	-0.03	0.54	0.96

**Mild injury (n=21)**	(Intercept)	261.93	33.38	< 0.01	78.66	31.30	0.01	32.25	0.97	< 0.01
	Pre5	-34.93	22.17	0.12	-9.89	14.17	0.49	-1.21	0.67	0.07
	Pre4	-34.75	20.75	0.10	-2.40	13.79	0.86	-1.13	0.64	0.08
	Pre3	-39.96	20.01	0.04^*^	-6.84	12.88	0.60	-0.94	0.62	0.13
	Pre2	-42.30	19.42	0.03^*^	-13.20	12.36	0.29	-0.91	0.59	0.13
	Post1	-52.70	19.71	0.01^*^	-11.61	12.80	0.37	-1.18	0.59	0.04^*^
	Post2	-7.09	20.38	0.73	2.89	12.90	0.82	-0.13	0.63	0.83
	Post3	-1.37	21.67	0.95	-2.49	13.72	0.86	-1.24	0.67	0.07
	Post4	-4.41	22.17	0.84	3.57	13.70	0.80	-0.88	0.65	0.18
	Post5	4.81	21.66	0.82	22.44	13.72	0.10	-1.06	0.65	0.11
	Days_off	-14.81	7.07	0.04^*^	0.14	6.95	0.98	-0.20	0.20	0.34

**Moderate injury (n=4)**	(Intercept)	84.04	53.68	0.13	78.31	75.34	0.31	26.50	2.40	< 0.01
	Pre5	24.35	46.44	0.61	-12.01	23.57	0.62	-0.71	1.52	0.65
	Pre4	70.82	45.88	0.14	-22.06	30.82	0.48	-2.21	1.51	0.16
	Pre3	6.11	40.48	0.88	-26.41	20.54	0.21	0.20	1.33	0.88
	Pre2	64.41	40.48	0.13	10.95	20.54	0.60	0.46	1.33	0.73
	Post1	-31.03	37.32	0.42	-30.09	18.81	0.13	0.38	1.22	0.76
	Post2	-37.27	37.32	0.33	-43.09	18.81	0.03^*^	-1.92	1.22	0.13
	Post3	8.34	37.32	0.83	-9.05	18.81	0.64	0.19	1.22	0.88
	Post4	-34.44	40.35	0.40	-6.26	20.49	0.76	-0.75	1.22	0.54
	Post5	46.19	37.32	0.23	-8.97	18.81	0.64	0.53	1.33	0.70
	Days_off	6.11	3.80	0.25	-0.23	5.93	0.97	0.33	0.18	0.21

**Severe injury (n=10)**	(Intercept)	197.71	38.36	< 0.01	106.73	50.12	0.04	29.87	1.16	< 0.01
	Pre5	6.92	34.73	0.84	-7.46	29.62	0.80	0.31	0.91	0.74
	Pre4	46.18	34.69	0.19	10.19	27.67	0.71	-1.11	0.89	0.21
	Pre3	53.96	32.31	0.10	27.33	26.74	0.31	0.60	0.89	0.50
	Pre2	4.50	32.20	0.89	5.53	27.02	0.84	-0.34	0.88	0.70
	Post1	18.96	32.23	0.56	26.53	27.26	0.33	0.87	0.95	0.37
	Post2	-5.04	33.45	0.88	-0.74	27.26	0.98	0.01	0.92	0.99
	Post3	4.69	33.45	0.89	3.52	27.26	0.90	0.81	0.92	0.38
	Post4	25.67	33.45	0.45	-6.43	27.26	0.81	0.79	0.92	0.39
	Post5	-5.43	34.81	0.88	-1.36	29.74	0.96	-0.06	0.96	0.95
	Days_off	-0.54	0.38	0.16	-0.28	0.53	0.59	0.01	0.01	0.67

Time windows represent 7-day periods relative to the injury. “Pre” periods refer to the weeks before the injury (e.g., Pre1 = 0–7 days before), and “Post” periods refer to the weeks after return to play (e.g., Post1 = 0–7 days after RTP). The intercept represents the Pre1 time window for a medical attention injury with 0 days of time loss. Metrics include High-Speed Running (HSR, > 21 km/h), Sprint Distance (> 24 km/h), and Maximum Velocity (V_max_, in km/h). Values are presented as mean ± Standard Error (SE).

## DISCUSSION

To the author’s knowledge, this is the first study to prospectively analyze (over 5 weeks) the return to play after both hamstring and quadriceps injuries during full microcycles in professional male football players. Previous studies [24–26] have analyzed differences between pre- and post-injury periods in competitive games but have not shown how entire weeks can be affected. The results show that hamstring injuries in football players require more time to achieve pre-injury values in physical performance variables.

Hamstring injuries are more likely to recur compared to quadriceps injuries in football. Studies consistently show that the recurrence rate for hamstring injuries is higher, with a significant percentage of athletes experiencing reinjury within a few months after returning to play [27], some findings indicate that hamstring injuries account for approximately 17% of all soccer-related injuries, with a recurrence rate of about 34% within the same season [28]. This highlights the importance of a careful and progressive rehabilitation approach to minimize the risk of reinjury.

To reduce the likelihood of hamstring reinjury, the introduction of load and exposure to competitive demands must be gradual. Reintroducing players to full competition should follow a progressive approach that takes into account the individual needs of each athlete, ensuring that physical and performance capacities are restored in a safe and controlled manner [29]. This gradual process allows for adequate adaptation to the stresses of the sport and lowers the risk of overloading muscles too soon.

The demands most associated with hamstring injuries are those related to high-intensity running and sprinting, particularly when accumulated over time. These activities put considerable eccentric load on the hamstrings, increasing the risk of strain [30]. Thus, players should gradually regain their previous performance values and their individual maximums before returning to full intensity and competitive levels [31].

A progressive return to official competition is essential for preventing reinjury. Players must progressively rebuild their strength, endurance, and sprinting capabilities to match or exceed their preinjury performance levels. Monitoring these parameters is crucial for ensuring that the player is physically prepared to withstand the demands of professional football.

The role of hamstrings during sprints and high-intensity velocity actions is crucial [32]. The present study shows that V_max_ decreases after the injury and does not recover to its previous values until the fourth week. This could be explained by the isokinetic deficit that most players present when returning to play after a hamstring injury [33, 34]. Furthermore, it has been shown that the length of the injury directly affects the decrement in maximal speed: the more days off, the lower the velocity reached after injury. These results concerning V_max_ and are similar to the findings of Mendiguchia et al. [16]. Additionally, [35] identified elevated HSR in the 5-min preceding injury as a predictor of hamstring injuries, reinforcing the importance of monitoring acute workload patterns in injury prevention strategies. This aligns with our findings showing reduced HSR in the immediate pre-injury period, suggesting that subtle changes in external load may precede injury events. Furthermore, emphasizes the dynamic interaction between acute and chronic loads in shaping injury risk, supporting the need for integrated monitoring frameworks that go beyond isolated metrics [36].

It is also notable that high-intensity movement patterns (HSR and sprint distance) revealed lower values in Pre1 compared to Pre2 and Pre3, especially in moderate and severe injuries. This could suggest that external workload may decrease prior to injury, even when demands remain the same. Thus, this reduction in workload could be influenced by fatigue, potentially increasing the risk of hamstring injury [30, 37].

External load should be considered a key criterion in the RTPerf process [38]. Although professional teams monitor training load, they must acknowledge that external load values are not fully restored when players return to competition. This incomplete recovery may increase the risk of reinjury, as players might not be adequately prepared to meet the demands of the game [39].

No significant findings were observed when exploring severe quadriceps injuries. External load remains stable before and after the injury, with no significant differences that can be explained. This highlights a significant difference in the RTP process concerning the external load of an injury depending on its location. When a player who suffered a hamstring injury needs more time to recover their pre-injury values, a player with a quadriceps injury will have similar variables [40].

It is known that the conditional demands are dependent on the demarcations of the game model and training model, but this is not a limitation in this study because each player is compared to himself, and because they are elite, it is unusual to find changes in game model or playing position. However, it is essential to acknowledge the limitations of the study. While this research provides valuable information, it must be noted that some injury observations have been missed due to lack of follow-up by staff. It is also important to consider that many players change team from one season to another, and coaching staff also undergo modifications. Therefore, a longitudinal study like this is clearly affected by these factors.

In this sense, future research should incorporate extended followup beyond 5-weeks to assess long-term outcomes, include neuromuscular testing to elucidate physiological mechanisms, and integrate validated psychological readiness assessments to better capture RTP and RTPerf potential.

## CONCLUSIONS

This study highlights the importance of considering the injury location (hamstrings or quadriceps) as well as its severity. Strength and conditioning coaches should note that maximal velocity is significantly reduced in severe hamstring injuries and does not return to pre-injury levels for at least five weeks. Furthermore, players with hamstring injuries exhibit a decline in HSR performance until the second week post-return. Specifically, severe hamstring injuries resulted in a 1.43 km/h reduction in Vmax and a 32.90 m decrease in HSR during the first week post-RTP, while mild quadriceps injuries led to a 1.18 km/h drop in V_max_ and a 52.70 m reduction in HSR. These findings underscore the importance of daily load monitoring and close coordination between rehabilitation and performance staff. Future research should include longer follow-up periods, neuromuscular testing, and psychological readiness assessments to better inform return-to-performance strategies.

## Access to study data

Due to privacy concerns of the club, the data supporting the findings of this study are available upon request. Interested researchers can obtain the data by contacting the corresponding author via email.
